# An RNA-based system to study hepatitis B virus replication and evaluate antivirals

**DOI:** 10.1126/sciadv.adg6265

**Published:** 2023-04-12

**Authors:** Yingpu Yu, William M. Schneider, Maximilian A. Kass, Eleftherios Michailidis, Ashley Acevedo, Ana L. Pamplona Mosimann, Juliano Bordignon, Alexander Koenig, Christine M. Livingston, Hardeep van Gijzel, Yi Ni, Pradeep M. Ambrose, Catherine A. Freije, Mengyin Zhang, Chenhui Zou, Mohammad Kabbani, Corrine Quirk, Cyprien Jahan, Xianfang Wu, Stephan Urban, Shihyun You, Amir Shlomai, Ype P. de Jong, Charles M. Rice

**Affiliations:** ^1^ Laboratory of Virology and Infectious Disease, The Rockefeller University, New York, NY 10065, USA.; ^2^ Department of Infectious Diseases, Molecular Virology, University Hospital Heidelberg, Heidelberg, Germany.; ^3^ Infectious Diseases Research Unit, GSK, Upper Providence, PA 19426, USA.; ^4^ Computational Biology, Genome Sciences, GSK, Stevenage, UK.; ^5^ German Center for Infection Research (DZIF), Partner Site Heidelberg, Heidelberg, Germany.; ^6^ Department of Physiology, Biophysics, and Systems Biology, Weill Cornell Medicine, New York, NY 10065, USA.; ^7^ Division of Gastroenterology and Hepatology, Weill Cornell Medical College, New York, NY 10065, USA.; ^8^ Department of Gastroenterology, Hepatology and Endocrinology, Hannover Medical School, Hannover, Germany.

## Abstract

Hepatitis B virus (HBV) chronically infects an estimated 300 million people, and standard treatments are rarely curative. Infection increases the risk of liver cirrhosis and hepatocellular carcinoma, and consequently, nearly 1 million people die each year from chronic hepatitis B. Tools and approaches that bring insights into HBV biology and facilitate the discovery and evaluation of antiviral drugs are in demand. Here, we describe a method to initiate the replication of HBV, a DNA virus, using synthetic RNA. This approach eliminates contaminating background signals from input virus or plasmid DNA that plagues existing systems and can be used to study multiple stages of HBV replication. We further demonstrate that this method can be uniquely applied to identify sequence variants that confer resistance to antiviral drugs.

## Teaser

An in vitro RNA-based system allows study of hepatitis B virus intracellular replication.

## INTRODUCTION

Hepatitis B virus (HBV) is a small DNA virus, transmitted by blood or other bodily fluids, which infects human hepatocytes. Upon HBV entry into hepatocytes, the relaxed circular, partially double-stranded DNA (rcDNA) genome is converted to the stable episomal form known as covalently closed circular DNA (cccDNA) (fig. S1A). cccDNA is transcribed by host RNA polymerase II (Pol II) to produce all HBV RNAs (fig. S1B), and cccDNA persistence in hepatocytes is thought to be responsible for chronicity (*1*, *2*).

Treatment options for chronic HBV infection are limited, and consequently, hepatitis B–related disease claims an estimated 820,000 lives each year (*3*). There is an effective vaccine to prevent HBV infection, but it does not benefit those already infected and vertical transmission remains a problem in some areas of the world. Standard therapies for chronic HBV include injectable interferon-α (IFN-α) and orally administered nucleot(s)ide analogs. IFN-α therapy can result in functional cure [HBV S antigen (HBsAg) loss] in ~10% of patients (*4*), but it often elicits intolerable side effects, and since few patients benefit, it is seldom used. In contrast, nucleoside/nucleotide analogs that effectively suppress HBV replication are well tolerated, but they do not eliminate cccDNA, and therefore, therapy is indefinite and often lifelong. New therapies are needed to improve HBV cure rates.

Lately, there has been renewed interest in curing chronic HBV. This has been driven, in part, by recent success in curing chronic hepatitis C virus infection and by the discovery of the HBV entry receptor, NTCP (*5*, *6*), making it possible to study the full HBV life cycle in cell culture. But despite progress, existing cell culture–based methods to study HBV have several limitations. For example, infection in vitro requires a high viral genome to cell ratio, which results in a high background of contaminating viral DNA and protein from the inoculum. Background contamination also plagues methods that initiate HBV replication with either plasmid DNA transfection or using cell clones with integrated HBV genomes. In these cases, much of the viral RNA and protein originate not from the authentic viral template, cccDNA, but from either the transfected plasmid or integrated DNA.

To address these challenges, we developed a method to initiate HBV replication with in vitro–transcribed pregenomic RNA (pgRNA). The idea to initiate HBV replication with pgRNA was supported by previous work on a relative of HBV, duck hepatitis B virus (DHBV), with the observation that DHBV pgRNA can initiate infection in cultured cells (*7*). We reasoned that initiating HBV replication and gene expression with in vitro–transcribed pgRNA would eliminate contaminating HBV DNA that confounds existing cell culture systems. Furthermore, since not all viral RNAs are required to initiate replication, some viral proteins (e.g., HBsAg, a commonly used marker for infection) would be produced only if the viral life cycle progressed and cccDNA was established. Here, we demonstrate that initiating HBV replication with in vitro–transcribed pgRNA can be used to study post-entry steps in the HBV life cycle with excellent signal-to-noise properties. These properties, for the first time in cell culture, enable identification of sequence variants that confer resistance to anti-HBV drugs.

## RESULTS

### In vitro–transcribed HBV pgRNA is infectious

**Fig. 1.**
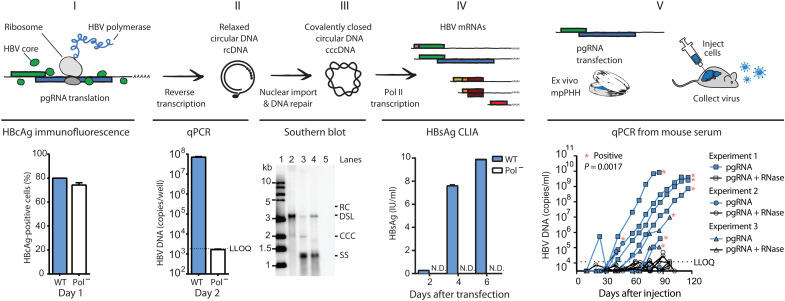
The RNA launch system. From left to right, the figure shows that in vitro–transcribed pgRNA is (I) translated (HBcAg is detected by immunofluorescence in ~80% of Huh-7.5-NTCP cells), (II) reverse-transcribed [qPCR detects HBV DNA and the signal is HBV polymerase dependent, as a catalytic site mutation (Pol^−^, YMHD) decreases the DNA signal by 10,000-fold], and (III) transported to the nucleus to form cccDNA (Southern blot confirms that cccDNA is produced; see below for lane description); (IV) cccDNA is transcribed to produce viral mRNA and protein [shown by HBsAg chemiluminescent immunoassay (CLIA)]; and (V) in vitro–transcribed pgRNA transfected into mpPHH and engrafted into humanized mice initiates productive virus infection, whereas ribonuclease A (RNase A)–treated pgRNA fails to initiate infection. Red asterisks indicate mice considered HBV-positive based on having at least two consecutive samples with rising HBV DNA levels above the lower limit of quantification (LLOQ) indicated on the graph. LLOQ for CLIA assay = 0.05 IU/ml, corresponding to less than 1% of the untreated control. Values plotted for HBcAg staining, qPCR, and HBsAg CLIA are *n* = 3 ± SEM. WT, wild type; IU, international units; N.D., not detected; copies per well, one well of a six-well plate; kb, kilobase; Southern blot: lane 1, 1-kb ladder; lane 2, WT HBV DNA; lane 3, WT HBV DNA heated to 85°C; lane 4, WT HBV DNA heated to 85°C and then linearized with Eco RI; lane 5, Pol^−^ HBV DNA; RC, relaxed circular DNA; DSL, double-stranded linear DNA; CCC, covalently closed circular DNA; SS, single-stranded DNA. For mouse experiments, *n* = 12 pgRNA; *n* = 10 pgRNA + RNase.

To test whether HBV pgRNA could initiate the virus life cycle, we produced 5′ capped and 3′ polyadenylated HBV pgRNA in vitro with T7 bacteriophage RNA polymerase (RNAP) and measured products of HBV replication following pgRNA transfection into cultured human hepatoma cells expressing the HBV entry receptor (Huh-7.5-NTCP). As shown in Fig. 1, transfected HBV pgRNA is (I) translated to produce the HBV core protein [HBV core antigen (HBcAg)], (II) reverse-transcribed to HBV rcDNA by the viral Pol, and (III) transported to the nucleus to form cccDNA, (IV) yielding HBsAg expression. Southern blot confirms that pgRNA gives rise to cccDNA, and transfection of pgRNA encoding a catalytically inactive Pol mutant (YMHD) demonstrates that the HBV DNA and HBsAg signal is replication-dependent and that there is minimal to no contaminating background signal from the input material (*8*). This “RNA launch” method can also be used to study HBV replication in other cell lines commonly used to study HBV, such as Huh-7 and HepG2-NTCP (fig. S2A). We initiated experiments with Huh-7.5 cells expressing the HBV receptor, NTCP, to provide an opportunity for virus spread; however, the percentage of HBsAg-positive cells was similar in Huh-7.5 and Huh-7.5-NTCP cells, indicating that there is minimal to no virus spread in cell culture in this time frame under these conditions (fig. S2B). Figure S2C compares the advantages and disadvantages of this pgRNA transfection method with other existing systems routinely used to study HBV.

The Southern blot in Fig. 1 indicated that while cccDNA is formed, most reverse-transcribed DNA is double-strand linear. To investigate this further, we treated DNA with plasmid-safe deoxyribonuclease (DNase), which degrades linear DNA but not rcDNA or cccDNA (fig. S3A) (*9*). These results demonstrate that rcDNA is formed, and recently, it was shown that removing two nucleotides from the 5′ end of the pgRNA construct that we used in our studies increases the ratio of rcDNA:dslDNA (double-stranded linear DNA) (*10*).

To establish proof of concept that HBV pgRNA can initiate the full virus life cycle, we transfected mouse-passaged primary human hepatocytes (mpPHHs) with HBV pgRNA in cell culture and then injected these cells into liver chimeric huFNRG mice in which most hepatocytes are human (*11*). We used huFNRG mice because, unlike cell culture systems, HBV replicates and spreads robustly in liver chimeras (*11*, *12*). Accordingly, in vitro–transcribed HBV pgRNA initiated a spreading infection in huFNRG mice (Fig. 1, right). To control for the possibility that residual DNA template used in the in vitro transcription reaction initiates infection rather than pgRNA, we included huFNRG mice that received mpPHHs transfected with ribonuclease A (RNase A)–treated pgRNA. As expected, none of the control mice became viremic. To further demonstrate that HBV pgRNA can produce infectious virus, we performed a Southern blot on hepatocytes extracted from one of the HBV-positive mice to confirm the presence of cccDNA (fig. S3B), and we inoculated naïve liver chimeric huFNRG mice with serum from this same mouse. All the naïve mice inoculated with this serum became viremic (fig. S3C). Together, these data demonstrate that 5′ capped and 3′ polyadenylated in vitro–transcribed HBV pgRNA alone, without additional RNA modifications, viral proteins, messenger RNAs (mRNAs), or noncoding RNAs, is sufficient to initiate the full HBV life cycle.

### Diverse HBV genotypes are amenable to the HBV RNA launch method

**Fig. 2.**
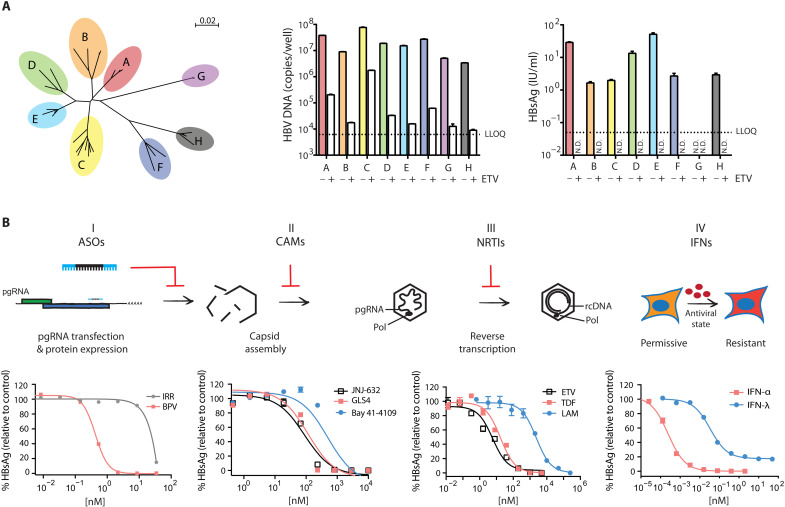
RNA launch method can be used to evaluate pan-genotype HBV replication and study multiple classes of anti-HBV drugs. (**A**) Left: Unrooted phylogenetic tree of HBV genotypes A to H. The tree was generated using SeaView (*46*). The scale bar indicates nucleotide substitutions per site. Middle: qPCR of intracellular HBV DNA for HBV genotypes A to H 2 days after transfection with and without 10 μM ETV. Right: HBsAg from HBV genotypes A to H was measured by CLIA 6 days after transfection with and without 10 μM ETV. For both qPCR and HBsAg CLIA, 3 ± SEM. (**B**) Left to right: The figure shows that in vitro–transcribed pgRNA can be used to test the efficiencies of (I) antisense oligonucleotides (ASOs), (II) capsid assembly modulators (CAMs), (III) nucleotide RT inhibitors (NRTIs), and (IV) interferons (IFNs). Secreted HBsAg was quantified by CLIA and normalized to controls at 4 days (ASOs) or 6 days (CAMs and NTRIs) after transfection with HBV genotype A pgRNA as described in Materials and Methods. Data plotted are *n* ≥ 3 for NRTIs and *n* ≥ 6 for other inhibitors. Error bars are ±SEM. LLOQ = 0.05 IU/ml, which corresponds to less than 1% of the untreated control. BPV, bepirovirsen; IRR, irrelevant ASO control; LAM, lamivudine; TDF, tenofovir disoproxil fumarate.

Until recently, most HBV cell culture studies have focused on genotypes A and D. Similarly, our initial work was performed with genotype A HBV. There are, however, additional HBV genotypes, several of which are prevalent in various parts of the world (Fig. 2A, left, and table S1) (*13*). It is increasingly recognized that studying multiple genotypes is important since they may differ in their clinical outcomes (*14*, *15*). Therefore, we next tested whether the RNA launch method could be readily applied to study additional HBV genotypes. We cloned representative pgRNA sequences for HBV genotypes B to H downstream of a T7 promoter and transfected plasmid DNA into cells to confirm that the HBsAg detection kit could recognize HBsAg from all eight HBV genotypes (fig. S4A). We then transfected cells with in vitro–transcribed pgRNA produced from these plasmids and found that all HBV genotypes yielded HBV DNA, and all but genotype G secreted detectable levels of HBsAg (Fig. 2A). Despite not secreting detectable levels of HBsAg, a low percentage of cells transfected with genotype G pgRNA stained positive for intracellular HBsAg (fig. S4B), in line with a previous report showing that HBsAg from HBV genotype G aggregates in the endoplasmic reticulum (*16*). For all genotypes, HBV DNA production and HBsAg secretion are replication-dependent since a reverse transcriptase (RT) inhibitor, entecavir (ETV), decreases both replication products. These results demonstrate that the RNA launch method can be applied to facilitate the study of post-entry steps for multiple HBV genotypes with low to no contaminating background signal.

### HBV RNA launch provides a convenient and sensitive method to evaluate HBV inhibitors

We next used the RNA launch method to evaluate a collection of HBV inhibitors that target different steps in the HBV life cycle. These include (i) bepirovirsen (BPV), an antisense oligonucleotide (ASO) currently in phase 2 clinical trials that targets HBV pgRNA and mRNAs; (ii) three capsid assembly modulators (CAMs); (iii) three RT inhibitors; and (iv) type I and III IFNs. Dose-response assays were performed in 96-well format with HBV pgRNA transfected in the presence of each inhibitor, and we quantified HBsAg in cell culture supernatants by chemiluminescent immunoassay (CLIA). All nine inhibitors tested reduced HBV replication in a dose-dependent manner, demonstrating that the RNA launch method can be used to study the efficacy of anti-HBV compounds and could potentially be used for high-throughput screens to identify inhibitors in a convenient and sensitive assay (Fig. 2B and table S2).

### HBV RNA launch can identify drug resistance mutations de novo

Drug-resistant viruses can arise when rare viral variants with a fitness advantage in the presence of drugs become enriched in the viral population. Identifying these variants can provide useful information during drug development and ongoing surveillance during treatment. For example, this information can be used to prioritize compounds and compound series for further development. It can also provide insight into virus-drug interactions and guide efforts to screen chemicals or improve the properties of candidate compounds. However, selecting drug-resistant variants for HBV in cell culture has been challenging since the virus spreads too poorly in vitro for drug-resistant variants to emerge. Consequently, there are no available cell culture–based methods to select antiviral resistance de novo for anti-HBV compounds. We hypothesized that the HBV RNA launch method could open an avenue to address this challenge.

**Fig. 3.**
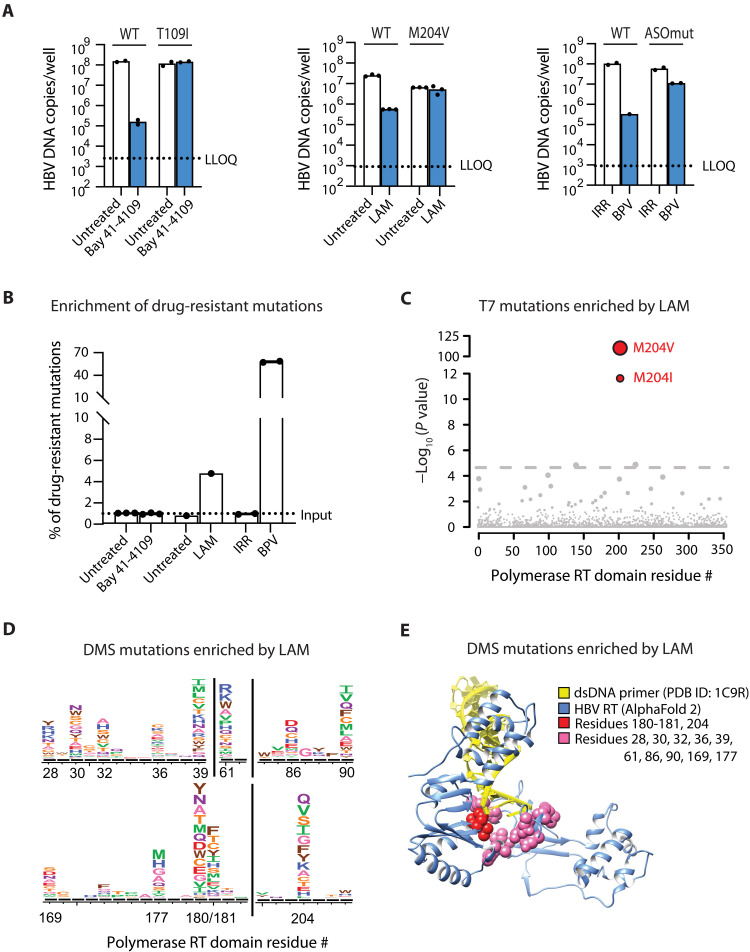
RNA launch method can be used to test drug efficiency and identify drug resistance mutations. (**A**) HBV DNA copy number by qPCR 2 days after transfecting cells with WT or drug-resistant pgRNA variants in the presence of antivirals. Left, 2.8 μM Bay 41-4109; middle, 400 μM LAM; right, 128:1 molar ratio ASO:pgRNA. Values plotted are mean with each replicate indicated as a separate dot. (**B**) Fold enrichment of drug-resistant HBV DNA 2 days after transfection. Drug-resistant pgRNAs were mixed with WT at 1:99 ratio with the same drug concentration described in (A). Values plotted are mean with each replicate indicated as a separate dot. (**C**) Sequencing HBV DNA from cells transfected with WT HBV pgRNA in the presence of 400 μM LAM identifies the two most common amino acid substitutions, i.e., M204V and M204I, found in LAM-resistant patients. Threshold = Bonferroni correction, alpha = 0.05. (**D**) Positively enriched amino acid substitutions were obtained from sequencing a deep mutational scanning (DMS) library covering the RT domain of HBV Pol in the presence of 400 μM LAM. (**E**) AlphaFold 2 model of HBV RT domain overlaid on a crystal structure of the HIV RT in complex with dsDNA (Protein Data Bank ID: 1C9R). The HIV protein is hidden. The three common amino acids found mutated in LAM-treated patients are colored red. Other amino acid positions positively enriched (arbitrary cutoff = 14-fold) are colored pink.

First, we asked whether HBV RNA launch could be used to study existing drug-resistant variants. We constructed two HBV pgRNAs containing known drug-resistant variants: a core mutant (T109I) known to confer resistance to the CAM, Bay 41-4109 (*17*), and a Pol mutant (M204V) known to confer resistance to the RT inhibitor lamivudine (LAM) (*18*). As expected, each of these mutant pgRNAs yielded high levels of HBV DNA in the presence of their respective inhibitors, while wild-type (WT) pgRNA was potently inhibited (Fig. 3A, left and middle). Since there are no known mutations that confer resistance to BPV, we engineered an HBV pgRNA construct (ASOmut) that contains eight nucleotide substitutions within the BPV binding site that should decrease activity (fig. S5A). These mutations do not alter the amino acid sequence of the Pol protein. We then compared the levels of WT or ASOmut HBV DNA 2 days after cotransfecting pgRNA with a 128-fold molar excess of BPV or an irrelevant (IRR) ASO control. In the presence of IRR, ASOmut HBV pgRNA yielded only slightly less HBV DNA than WT, whereas ASOmut pgRNA was ~10- to 100-fold less sensitive to BPV than WT pgRNA (Fig. 3A, right). These results demonstrate that the HBV RNA launch approach can be applied to evaluate the impact HBV variants have on the in vitro efficacy of various anti-HBV inhibitors.

Ideally, to discover variants that confer drug resistance, one could enrich drug-resistant HBV variants from a population. Limitations of existing cell culture HBV systems have thus far precluded this application, but given the low background of input DNA characteristic of RNA launch, we hypothesized that it should be possible to enrich drug-resistant variants that are selectively reverse-transcribed in the presence of drug. Toward this goal, we transfected a mixture of pgRNAs containing 99% WT sequence and 1% drug-resistant sequence, and then sequenced HBV DNA 2 days after transfection. We found that the LAM-resistant M204V mutant was enriched ~5-fold in the presence of LAM, and the ASOmut sequence was enriched ~60-fold in the presence of BPV (Fig. 3B). In contrast, we were unable to enrich the Bay 41-4109–resistant T109I variant from a mixed population, indicating that this method can enrich drug-resistant variants for some but not all targets (see Discussion).

Last, we tested whether we could enrich rare drug-resistant HBV variants de novo from a diverse population of pgRNAs. We used LAM for this proof-of-concept experiment, and the selection strategy is outlined in fig. S5B. To generate a diverse population of pgRNA, we first relied on the natural error rate of T7 RNA Pol to generate heterogeneity in HBV pgRNA from the highly uniform plasmid DNA template. Using the CirSeq method to characterize our input in vitro–transcribed HBV pgRNA, we observed a mutation frequency like the previously reported error rate of T7, ~10^−4^ (fig. S5C) (*19*, *20*). Two days after transfecting LAM-treated Huh-7.5-NTCP cells in triplicate with pgRNA, we harvested DNA, amplified the HBV genome by polymerase chain reaction (PCR) (fig. S5D), and sequenced the region encoding the RT domain. We found that the two most common clinically relevant LAM resistance mutations, i.e., M204V and M204I, were enriched (Fig. 3C) (*18*). This established that the RNA launch method could be used to identify drug-resistant HBV variants de novo.

Additional drug resistance mutations arise in LAM-treated patients, such as L180M and A181S/T/V, which were not enriched in our data. This could be due to the low frequency of variants in our input pgRNA, and we wondered if additional LAM resistance mutations would appear if our input pgRNA population were more diverse or if variants were present at a higher frequency. To increase diversity in our input pgRNA, we used deep mutational scanning (DMS) to generate a plasmid library where every codon in the RT domain of HBV Pol was randomized such that most plasmids in the library harbor a single amino acid mutation (*21*). The DMS library of the HBV RT domain yielded approximately 10-fold more diversity at each nucleotide position compared to the diversity generated by T7 RNAP (fig. S5, C and E). We used this plasmid library as the template to in vitro–transcribe HBV pgRNAs, and we used a similar selection strategy as the prior LAM treatment experiment (fig. S5B). Using the DMS library as input, we obtained many more positively enriched sequence variants in the reverse-transcribed DNA population (Fig. 3D). In addition to the M204V and M204I variants that we previously identified, the DMS library enabled us to identify additional common LAM-resistant variants including mutations at position L180 and A181.

We found that many mutations are enriched at positions 180, 181, and 204, beyond those identified in LAM-treated patients. Furthermore, we also enriched variants at other positions in RT that were not previously associated with LAM resistance. To visualize where these mutations are in three dimensions, we generated a structural model of the HBV Pol RT domain using AlphaFold 2 and overlaid this model onto a crystal structure of HIV RT in complex with a double-stranded DNA (dsDNA) primer (Protein Data Bank ID: 1C9R) (Fig. 3E). The structural model annotated with both clinically identified and DMS-identified LAM resistance variants reveals spatial clustering of these mutations. On the basis of homology to HIV RT (fig. S6), some residues in the HBV RT cluster at positions spatially analogous to LAM resistance mutations found in HIV RT and may interact with incoming nucleotides (*22*).

### HBV RNA launch provides mechanistic insights into an ASO targeting HBV RNA

Compared to RT inhibitors that act on the Pol protein, the ASO BPV acts by a fundamentally different mechanism—by targeting HBV mRNAs for cleavage. We next applied the RNA launch method to assess the impact of HBV variants on BPV’s antiviral activity and to potentially gain insight into its mechanism of action.

**Fig. 4.**
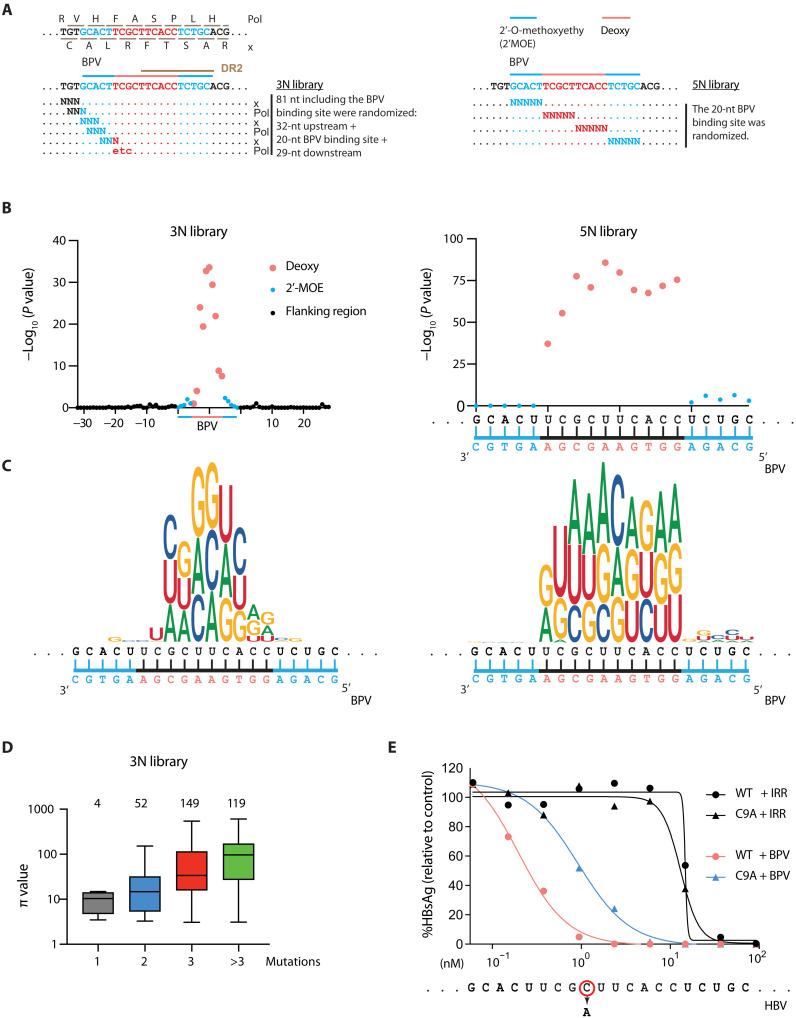
Nucleotide enrichment pattern in HBV DNA following BPV treatment. (**A**) Design of the 3N (left) and 5N (right) deep mutation scanning library used to enrich BPV-resistant HBV sequences. Colors indicate nucleotide modifications in BPV: red, unmodified gap DNA region; blue, 2′MOE-modified RNA wings. Direct repeat 2 (DR2) is indicated with a brown line. (**B**) Positional enrichment of nucleotide mutants in the 3N (left) and 5N (right) libraries when comparing BPV-treated samples to IRR-treated samples. Each dot represents the combined *P* value for all three non-WT nucleotides at a given position. (**C**) Logo plot describes the nucleotide composition at each site. (**D**) π values for HBV individual sequences with the number of mutations per sequence binned as indicated on the *x*-axis. The total number of sequences in each bin with π ≥ 3 is indicated on top. (**E**) Dose-response curve of inhibition for WT and C9A mutant pgRNA in the presence of BPV or IRR ASOs. HBsAg was measured by CLIA 4 days after transfection and normalized to untreated control. R square and 95% confidence intervals are presented in table S2.

BPV has a typical “gapmer” ASO design—it contains 10 nucleotides (nt) of DNA flanked by 5-nt 2′-O-methoxyethyl ribose (MOE)–modified RNA “wings” (Fig. 4A, top right). The 2′MOE modifications in the wings increase binding affinity and resistance to nuclease degradation, and this ASO design acts primarily by recruiting cellular RNase H to cleave the DNA/RNA hybrid formed within the central gap region (*23*, *24*).

To gain insight into sequence requirements for BPV activity, we constructed two HBV pgRNA DMS libraries with randomized nucleotides: 3N and 5N libraries (Fig. 4A). The 3N library was designed on the basis of codons in the HBV Pol and HBx open reading frames and extends from 32-nt upstream to 29-nt downstream of the BPV binding site. The 5N library mutagenizes only the 20-nt BPV binding site in blocks of five nucleotides. Because of the method of library construction, some HBV pgRNAs in the population will contain multiple 3N or 5N mutations (*21*). As a result, the plasmid libraries contain many more unique sequences than predicted if only one site is mutated (table S4). Sufficient deep sequencing demonstrated that these libraries contain more than 95% of all expected variants, and all sequences were retained for our downstream analysis (fig. S7A).

We premixed pgRNAs in vitro–transcribed from either of these libraries with IRR or BPV and transfected these mixtures into Huh-7.5-NTCP cells. We then deep-sequenced the reverse-transcribed HBV DNA to identify variants enriched in the BPV-treated samples. The strong selective pressure exerted by BPV in both libraries is indicated by the loss of HBV sequence diversity in BPV-treated cells. In contrast, cells treated with IRR control retained many more variants at high abundance, as expected if only nonfunctional or unfit HBV variants are purged from the library (fig. S7A). Nucleotide positions of variants enriched in BPV-treated samples reveal a notable preference for mutations at the center “gap” region of the BPV binding site, with a sharp decline in enrichment for bases located in the flanking “wing” regions (Fig. 4B). There is no apparent sequence preference other than the fact that the sequence should be non-WT, and the results indicate that G:U base pairs in the gap region also negatively affect activity (Fig. 4C).

Aside from the importance of the position of the mutated nucleotide, we also expected that the number of mutated nucleotides in each pgRNA would correlate with its level of enrichment. We used π-values, which combines *P* value and fold enrichment (*25*), to rank unique variants (table S5), and using π ≥ 3 as an arbitrary cutoff for inclusion, we found that the degree of enrichment positively correlates with the number of mutations in the pgRNA (Fig. 4D and fig. S7B).

The BPV binding site is located within a highly conserved region of the HBV genome, which encodes Pol and HBx and overlaps with direct repeat 2 (DR2), which is required to produce plus-strand HBV DNA. Despite these sequence constraints, single-nucleotide variants have been identified in the BPV binding site in clinical samples, such as a C-to-A mutation that appears in approximately 1% of genotype A sequences in an online HBV sequence database [C9A based on numbering within the BPV binding site (fig. S8 and table S6)]. This single-nucleotide mutant is one of many enriched in our BPV-treated samples. To confirm that the C9A variant provides some BPV resistance in our assay, we cloned and tested this variant in a dose-response assay and observed an approximate 4.7-fold shift in the IC_50_ (median inhibitory concentration) of C9A relative to WT (Fig. 4E and table S2). These results agree with our library screening results and indicate that C9A confers partial resistance to BPV treatment in vitro. The clinical relevance of such variants will be further investigated.

## DISCUSSION

Here, we report a cell culture–based method to study HBV, which has several unique advantages compared to other commonly used approaches. One of the main advantages is the near absence of HBV DNA and HBsAg signal from input material. This creates opportunities to study HBV replication in medium- and high-throughput formats using simple readouts such as quantitative PCR (qPCR) and CLIA with fewer sample processing steps than are typically required when using infection or plasmid transfection–based approaches (*26*). A limitation of this approach is that initiating replication with pgRNA bypasses viral entry, so it is not suitable for studying early events in HBV infection including receptor binding, internalization, and formation of cccDNA from incoming viral DNA. However, the method can be used to study how genotype-specific differences or mutations affect post-entry steps such as pgRNA packaging, reverse transcription, formation of replication intermediates, cccDNA formation from intracellular rcDNA, and gene expression.

As a result of the low DNA background, a major technical advance achieved by this method is the ability to study HBV sequence variants in pooled, high-throughput assays and identify drug resistance mutations de novo. However, not all anti-HBV compounds are candidates for this application. The method is not suitable for studying monoclonal antibodies targeting HBsAg or other inhibitors targeting virus entry. In addition, while some anti-HBV compounds, such as CAMs, can be studied with this RNA launch assay, we were unable to enrich CAM-resistant variants from a mixed population of sequences. We believe that this is because transfection delivers many pgRNAs per cell, and only variants that retain a genotype-phenotype link will appear enriched in the reverse-transcribed HBV DNA. It is likely that pgRNAs encoding CAM-resistant mutations are not selectively reverse-transcribed because CAM-resistant core proteins have little or no preference for encapsidating pgRNAs harboring CAM-resistant mutations. In other words, core proteins produced from one pgRNA can interact with core proteins produced from another pgRNA in the same cell, and genetic linkage is therefore lost. In contrast, we were readily able to enrich variants that confer resistance to LAM because HBV Pol has a cis-preference for binding, packaging, and reverse transcribing the pgRNA from which it was translated (*27*). Consequently, pgRNAs that encode LAM-resistant Pol are selectively reverse-transcribed in the presence of the drug. Likewise, it was possible to enrich BPV-resistant variants because these pgRNAs can evade RNase H cleavage and be reverse-transcribed regardless of what happens to other pgRNAs in the same cell. Together, we conclude that, with the current method, mutants that affect cis-acting functions can be efficiently enriched from a population, whereas the ability to enrich mutations that affect trans-acting functions will require further method development.

Using the DMS approach in LAM-treated samples, we identified many mutations in HBV Pol in addition to the mutations commonly observed in the clinic. We suspect that some of these mutations are not observed clinically because they require multiple mutations within a codon, which will be less frequent in a natural infection than in our DMS libraries. Another possibility is that many of the mutations that we enriched in the presence of LAM may be less fit in a natural infection than in our cell culture–based assay. For example, the entire HBsAg protein is encoded by the same nucleotides that encode Pol in an overlapping reading frame. As a result, most mutations in Pol will also mutate HBsAg. In a natural infection, selection on HBsAg will constrain Pol variation, but these constraints are absent in our assay and likely enabled us to obtain a more comprehensive profile of LAM resistance mutations. The fact that structurally analogous LAM resistance mutations have been identified in the HIV RT (*22*), which is not constrained by an overlapping reading frame, supports this hypothesis.

Similarly, we obtained a more comprehensive view of the sequence requirements for BPV activity than would be possible in other systems. For example, we detected many enriched mutations in the BPV binding site, although this is one of the most conserved regions in the HBV genome that encodes both Pol and HBx and overlaps with DR2. We were likely able to enrich a variety of mutations in this region because HBx is not required for reverse transcription (*28*), and while DR2 is important for the proper formation of rcDNA, it is not necessary to produce minus-strand DNA (*29*). Our HBV DNA amplification protocol does not distinguish full-length minus-strand DNA from rcDNA or dslDNA. Consequently, some mutations that confer BPV resistance are unlikely to be compatible with efficient virus replication. This result does not detract from the utility of this method to cast a wide net and screen thousands of variants to identify and prioritize a smaller number of mutants for further investigation.

These results can also provide mechanistic insight into the function of BPV and potentially other ASOs. We observed a notable difference in the impact of mutations in the outer wing regions containing 2′MOE-modified RNA bases compared to the non–2′MOE-modified DNA gap region. These results highlight the gap as a region of potential concern for the emergence of resistance, while mutations in the wings may be less consequential. This aligns with previous research indicating that this ASO configuration works predominantly by recruiting cellular RNase H and that the 2′MOE bases in the wing regions increase thermal stability but are resistant to cleavage (*30*).

One interesting observation from our data is that G:U base pairs are not well tolerated in the gap region. It was previously reported that G:U base pairs are tolerated in steric-blocking RNA ASOs uniformly modified with 2′MOE (*31*). The fact that they are not well tolerated in the context of BPV and HBV suggests that G:U base pairs in DNA/RNA hybrids may negatively affect RNase H cleavage. Another interesting observation is the centric pattern of resistance. This may be driven by binding affinity or the impact of mismatches on RNase H cleavage. The 2′MOE-modified wing regions are resistant to RNase H cleavage; nevertheless, mismatches in the wings should presumably have some negative impact on binding. The fact that mismatches in the wings appear to be of little consequence may stem from the fact that in our experimental design, the ASO is cotransfected with the HBV pgRNA. This prebinding scenario likely presents a more optimal condition for annealing than in a complex cellular environment. It is possible that the impact on binding affinity is underrepresented with this protocol. In any case, we suspect that it will hold true that the gap region will remain sensitive to mutations, and the wings will be more tolerant.

From a clinical perspective, identifying single and double mutations that confer partial resistance to BPV is relevant, as these mutations could potentially lead to the emergence of more resistant or fit variants. Our study found that the 3N library outperformed the 5N library in detecting single and double mutations, despite the presence of many of the same mutants in the 5N library. We hypothesize that this results from differences in library dynamics. The higher percentage of BPV-resistant variants in the 5N library may diminish our ability to rank less-resistant variants. If this hypothesis is correct, then a 1N library or well-designed 2N library may be better suited for investigating pathways to resistance against ASOs that could emerge in a clinical setting.

Although the BPV binding site is highly conserved, there are several polymorphisms found at low frequency (~1%) in clinical isolates (*32*). One example is a C-to-A mutation immediately upstream of DR2. We found that mutations at this position were enriched in our library screens even when we restricted the analysis to include only sequences harboring a single mutation. Subsequent dose-response experiments with this mutant further support the library screening results and indicate that single polymorphisms within the center of the gap region of the BPV binding site can confer partial BPV resistance.

In conclusion, we describe a unique and versatile tool to study basic HBV biology by initiating the virus life cycle with in vitro–transcribed pgRNA. The method can potentially be applied in high-throughput assays to discover anti-HBV inhibitors. In addition, by making it possible to screen thousands of HBV sequence variants at once in a pooled assay, it is possible to gain insights into drug resistance mechanisms and highlight variants of potential interest or concern that can be prioritized for more detailed evaluation with complementary lower-throughput methods. Together, this platform has a unique place in drug development pipelines and great potential to facilitate the discovery of HBV biology.

## MATERIALS AND METHODS

### Plasmids

All HBV genotype plasmid sequences used in this study have been uploaded to GenBank (table S1). Briefly, HBV pgRNA sequences were cloned into the pGEM-3Z plasmid backbone immediately downstream of a T7 promoter. The genotype A sequence has been previously described as serotype adw2 (*33*). Genotype D was derived from the HepDE19 cell line (*34*). Genotypes B, C, and E to H were derived from overlength HBV constructs originally cloned by Schaefer (*35*), and then subcloned into the pGEM-3Z plasmid backbone. The HBV-YMHD Pol mutant was made in the genotype A backbone (GenBank: MN172185).

The DMS library for the RT domain was constructed using the genotype A plasmid backbone. Mutagenesis was performed using PCR as described by Bloom (*36*). Briefly, we generated a list of codon tiling primers using a Python script written by the Bloom laboratory (https://github.com/jbloomlab/CodonTilingPrimers). This protocol randomly mutates each codon to all possible codons (NNN). We ordered all mutagenic forward and reverse primers from IDT in deep-well 96-well plates. All mutagenic reverse primers were combined at equimolar ratio and used together with forward primer, RU-O-18747, for 10 cycles using the enzyme and cycling conditions described by Bloom. Similarly, all mutagenic forward primers were combined at equimolar ratio and used together with reverse primer, RU-O-22160. The products of these two PCRs were used as input for 20 cycles of the joining reaction. The resulting PCR product was purified using Zymo Clean and Concentrator-5 (Zymo, catalog no. D4004), digested with Eco RI–HF (NEB, catalog no. R3101S) and Sac II (NEB, catalog no. R0157S), gel-extracted with agarose dissolving buffer (ADB) (Zymo, catalog no. D4001-1-50), and again purified with a DNA Clean & Concentrator-5 column. This digested product was ligated into the Eco RI–HF + SacI I–digested genotype A backbone with T4 DNA ligase and transformed into XL1-Blue electrocompetent bacteria (Agilent, catalog no. 200228). Bacterial colonies were grown overnight at 37°C on three 245 mm × 245 mm LB-carbenicillin plates. The following day, colonies were scraped, and DNA was extracted using the Qiagen HiSpeed Plasmid Maxi Kit (catalog no. 12663). The library contained more than 4 million transformants equal to roughly 185× coverage for each possible codon variant.

The 3N and 5N DMS libraries to mutagenize the BPV binding site and flanking sequence were constructed similarly, with several exceptions. For one, we set the minimum length of each primer as 32 nt to avoid overly short primers (we found in other DMS libraries that regions mutagenized with short primers yielded less diversity). For the 3N library, we mutagenized codons in both the HBV Pol and HBx reading frames. We ordered all mutagenic forward primers from IDT as one oligo pool (oPool) and all mutagenic reverse primers as a second oPool. We designed the 5N library primers manually and ordered four forward primers and four reverse primers as the third and fourth oPools separately. The flanking forward and reverse primers for both libraries were RU-O-26266 and RU-O-28077, respectively. The mutagenized PCR products were digested with Nco I–HF (NEB, catalog no. R3193L) and Not I–HF (NEB, catalog no.R3189L), ligated with T4 DNA ligase (NEB, catalog no. M0202M) into the gtA plasmid, and transformed into XL1-Blue electrocompetent bacteria (Agilent, catalog no. 200228). Each library contained more than 2 million transformants.

### Cells

Huh-7 and Huh-7.5 cells were maintained in Dulbecco’s modified Eagle’s medium (DMEM; Fisher Scientific, catalog no. 11995065) supplemented with 0.1 mM nonessential amino acids (NEAAs; Fisher Scientific, catalog no. 11140076) and 10% fetal bovine serum (FBS; HyClone Laboratories, lot. #AUJ35777). Huh-7.5-NTCP cells were made as previously described for HepG2-NTCP (*37*) and maintained as above. HepG2-NTCP cells were maintained on collagen-coated plates in DMEM supplemented with 10% FBS and 0.1 mM NEAA. mpPHHs were purified, plated, and cultured as previously described (*11*). The human biological samples were obtained commercially and were deidentified. They are exempt from National Institutes of Health (NIH) human subjects research under category 4, and their research use was approved by the Rockefeller University Institutional Review Board.

### Mice

*Fah*^−/−^ mice (*38*) were provided by M. Grompe (Oregon Health and Science University, Portland, OR) and crossed to nonobese diabetic *Rag1^−/−^ Il2rg^null^* mice (The Jackson Laboratory) to derive FNRG mice as previously described (*39*). FNRG mice were maintained on an ad libitum chow diet with amoxicillin and drinking water containing nitisinone (16 mg/ml; Yecuris, catalog no. 20-0028). To create human liver chimeras, FNRG mice received two intraperitoneal injections of retrorsine (Sigma-Aldrich, catalog no. R0382) (*11*) and were cycled off the drug nitisinone before intrasplenic transplantation with primary human hepatocytes HUM4188 (Lonza) as described previously (*39*). Mice were then cycled off nitisinone drinking water. Humanization was monitored by human albumin quantification in mouse serum using a human-specific enzyme-linked immunosorbent assay (Bethyl Laboratories, catalog no. E88-129). Humanized FNRG mice with human albumin values greater than 1 mg/ml were used for the HBV pgRNA inoculation experiment. To inoculate humanized FNRG mice with HBV pgRNA, pgRNA-transfected mpPHHs were mobilized 4 to 5 days after transfection and ~1.5 × 10^4^ cells were injected intrasplenically (*39*). To inoculate humanized FNRG mice with HBV from serum, each mouse was injected intravenously with 5.5 × 10^6^ HBV genome equivalents. To monitor viremia, DNA in mouse serum was extracted using the QIAamp DNA Blood Mini Kit (Qiagen, catalog no. 51106). Total HBV DNA was determined by quantitative PCR using TaqMan Universal PCR Master Mix with primers and probe listed in table S3. All experiments were conducted under animal use protocol 21056-H approved by the Rockefeller University Institutional Review Board.

### Synthesis of HBV pgRNA

Ten micrograms of plasmid DNA was linearized by digestion with Not I–HF. Linearized DNA was purified with the MinElute PCR Purification Kit (Qiagen, catalog no. 28004) according to the manufacturer’s instructions and diluted to 0.5 μg/μl in elution buffer (EB), and 2 μg was used as a template for in vitro transcription. Transcription was performed using the Ribomax T7 Kit (Promega, catalog no. P1320). The reaction was incubated at 37°C for 30 min followed by 15-min DNase (from kit) treatment at 37°C. To reduce the carryover of residual undigested plasmid or DNA fragments, the DNase-treated RNA was transferred to a new tube before purifying RNA using the RNeasy Mini Kit (Qiagen, catalog no. 74014) following the manufacturer’s instructions. The optional on-column DNase digestion step (Qiagen, catalog no. 79254) was included. Capping and polyadenylation were performed using T7 mScript Standard mRNA Production System (CELLSCRIPT, catalog no. C-MSC100625) following the Cap 1 mRNA protocol described in the user’s manual, and the RNA was again purified using the RNeasy mini kit without on-column DNase digestion. The expected RNA yield is ~45 to 60 μg per reaction.

### RNA transfection

Huh-7, Huh-7.5, and Huh-7.5-NTCP cells were seeded at 2.5 × 10^5^ cells per well in six-well plates or 5.0 × 10^4^ cells per well in 24-well plates. The medium was changed to 2 or 1 ml of DMEM containing 1.5% FBS and 0.1 mM NEAA for 6 well or 24 well just before transfection. For each transfected well (six-well plate), 0.5 μg of HBV pgRNA was mixed with 5 μl of Lipofectamine 2000 (Fisher Scientific, catalog no. 11668019) in 500 μl of Opti-MEM Reduced-Serum Medium (Fisher Scientific, catalog no. 51985034) and incubated at room temperature for 20 min. For 24-well plates, 0.1 μg of HBV pgRNA was mixed with 0.5 μl of Lipofectamine 2000 in 50 μl of Opti-MEM Reduced Serum Medium. The mixture was then added to cells and spinoculated by centrifugation at 1000*g* for 30 min at 37°C. Six hours later, the medium was removed and replaced with DMEM containing 10% FBS and 0.1 mM NEAA. For experiments designed to quantify HBsAg, fresh medium was supplemented with 2% dimethyl sulfoxide (DMSO). Conditions for 96-well plates for drug titrations are described below.

HepG2-NTCP cells were transfected similarly, with the following exceptions: 1 × 10^5^ cells were seeded per well in collagen-coated 24-well plates, and no DMSO was added after transfection.

Cultured mpPHHs (1.1 × 10^6^ to 1.3 × 10^6^) were seeded per well in six-well plates 3 days before transfection in W10 medium (*11*). Medium was removed and replaced with hepatocyte culture medium (HCM) (Lonza, catalog no. CC-3198) 1 day later. Fresh HCM was replaced every 2 days before transplantation into mice, as described above.

### DNA transfection

To detect HBsAg produced by plasmid transfection from genotypes A to H (table S1), Huh-7.5-NTCP cells were seeded at 2.5 × 10^5^ cells per well in six-well plates and transfected 2 days later in DMEM containing 1.5% FBS and 0.1 mM NEAA with 2.5 μg of plasmid DNA using X-tremeGENE 9 DNA Transfection Reagent (Sigma-Aldrich, catalog no. 6365779001) at 5:1 reagent:DNA ratio. Supernatants were collected 4 days after transfection for HBsAg CLIA.

### Analysis of HBV translation and replication

HBsAg was quantified using a CLIA kit (Autobio Diagnostics Co., Zhengzhou, China) according to the manufacturer’s instructions or by flow cytometry. For flow cytometry analysis, cells were harvested 6 days after transfection, fixed and permeabilized with BD Cytofix/Cytoperm (catalog no. BD554714) following the manufacturer’s instructions, and stained with human monoclonal anti-HBs H-20 at 0.5 ng/μl (*12*). HBsAg experiments were performed in a 24-well format. To quantify HBV DNA, total DNA was extracted from individual wells of 6- or 24-well plates using the QIAamp DNA Blood Mini Kit (Qiagen, catalog no. 51106) and HBV DNA was detected by qPCR as previously described (*37*). In addition, we designed a separate set of primers targeting the HBV core region of genotype A that we used in SYBR Green assays. Both assays gave similar results; however, the SYBR assay more consistently yielded a background signal (YMHD, Pol mutant) below the limit of quantification. Primers and probe sequences for qPCR are listed in table S3. In instances where the primer binding sites differed among genotypes, genotype-specific standard curves were generated using plasmid dilutions. For immunofluorescence analysis, cells were stained with Anti-HBc (Austral Biologicals, catalog no. HBP-023-9) at 1:500 dilution. Fluorescent images were quantified in ImageJ (NIH, Bethesda, MD) using the thresholding method, like what has been previously described (*40*). Southern blot was performed by combining two protocols previously described (*41*, *42*) with several modifications. HBV cccDNA was enriched by Hirt extraction as previously described (*41*). The extract was digested at 37°C for 2 hours in 400 μl of CutSmart buffer (NEB) with 40 μg of RNase A (Thermo Fisher Scientific, catalog no. EN0531) to remove RNA and 60 U of Hind III–HF (NEB, catalog no. B7204S) to digest genomic DNA. The reaction was stopped by adding 200 μg of proteinase K (Fisher Scientific, catalog no. 25530015) for 30 min at 37°C followed by phenol/chloroform extraction. DNA was precipitated with ethanol and dissolved in 25 μl of TE [10 mM tris, 1 mM EDTA (pH 8)]. For plasmid safe DNase (PSD) treatment, 25 μl of Hirt-extracted DNA was digested for 8 hours at 37°C with 50 U of PSD in 250-μl final volume containing 1 mM adenosine triphosphate (ATP). The reaction was stopped by adding phenol, and nuclease-resistant DNA was recovered by ethanol precipitation. DNA extracted 2, 4, and 6 days after transfection was combined and split into three separate tubes for further treatment with heat and/or Eco RI digestion. Samples were run overnight at 4°C in a 1.2% agarose tris-acetate-EDTA (TAE) gel and then transferred to a Hybond-XL membrane (Fisher Scientific, catalog no. RPN303N) for 36 to 48 hours. ^32^P-labeled hybridization probes were prepared with the Prime-It II Random Primer Labeling Kit (Agilent Technologies, catalog no. 300385) using linearized 2× HBV DNA as a template as previously described (*42*). Probes were hybridized overnight at 65°C. The membrane was washed as described (*41*), and HBV DNA was visualized by phosphorimager.

### CirSeq analysis of in vitro–transcribed pgRNA

CirSeq of in vitro–transcribed pgRNA of HBV genotype A was performed as previously described (*20*). Briefly, fragmented pgRNA was circularized and converted to DNA by rolling-circle reverse transcription, yielding tandemly repeated cDNAs. Two replicates were prepared and 325-cycle single-end sequencing of both replicates was performed on an Illumina MiSeq at the Rockefeller University Genomics Resource Center. Tandem repeat reads were converted to consensus sequences, filtering out random errors generated during library preparation and sequencing, and then mapped to the reference. The error rate of T7 Pol was determined as the frequency of mismatches with respect to the reference sequence for bases with an average quality score of ≥20. Sequences are deposited in the National Center for Biotechnology Information (NCBI) Sequence Read Archive, BioProject accession number PRJNA557489.

### HBV inhibitor experiments

CAMs were purchased from MedChemExpress: JNJ-632 (catalog no. HY-112564), GLS4 (catalog no. HY-108917), and Bay 41-4109 (catalog no. HY-100029).

#### Reverse transcriptase inhibitors

ETV was purchased from Cayman Chemical Company (catalog no. 209216-23-9), LAM was purchased from Sigma-Aldrich (catalog no. L1295), and tenofovir disoproxil fumarate was obtained through the AIDS Reagent Program, Division of AIDS, National Institute of Allergy and Infectious Diseases, NIH.

#### Interferons

Human IFN-α2a was purchased from PBL Assay Science (catalog no. 11101-2), and human IFN-λ3 was purchased from R&D Systems (catalog no. 5259-IL-025).

#### Antisense oligos

BPV (5′-GCAGAGGTGAAGCGAAGTGC-3′) and IRR control (5′-CCTTCCCTGAAGGTTCCTCC-3′) were obtained from GSK.

For CAM, RT inhibitor, and IFN experiments, Huh-7.5-NTCP cells were seeded at 1 × 10^4^ cells per well in 96-well plates 2 days before transfection. One day before transfection, medium was changed and replaced with 100 μl of DMEM containing 10% FBS and 0.1 mM NEAA with HBV inhibitors or vehicle control. The following day, each well was transfected as described above with 17 ng of HBV pgRNA in 100 μl of DMEM containing 1.5% FBS and 0.1 mM NEAA. Six hours later, the medium was changed to 100 μl of DMEM containing 10% FBS and 0.1 mM NEAA. Every 2 days, the medium was collected and replaced and 50 μl of collected supernatants was used for HBsAg CLIA at day 6, as described above. For CAMs and nucleotide RT inhibitors (NRTIs), drugs were added 1 day before transfection and maintained in the medium throughout the experiment. Cells were treated once with IFNs 14 to 16 hours before transfection, removed during transfection, and not replaced.

For ASO experiments, Huh-7.5-NTCP cells were seeded at 1.2 × 10^4^ cells per well in 96-well plates 2 days before transfection. The pgRNA was premixed with ASO at molar ratios specified in the figures. Supernatants were collected for HBsAg CLIA at day 4. Cells were collected at day 2 for HBV DNA isolation. HBsAg CLIA values were normalized to untreated conditions or the average values for the three lowest concentrations of ASO if the curve was at a plateau. Dose-response analysis was performed in Prism using three-parameter curve fitting for data in Fig. 2B and four-parameter curve fitting for data in Fig. 4E.

### Enriching LAM-resistant viral variants produced by T7 polymerase error

To enrich T7 Pol–produced mutations that confer LAM resistance, 2.5 × 10^5^ Huh-7.5-NTCP cells were seeded 2 days before pgRNA transfection as described above. The cells were treated with 400 μM LAM 24 hours before RNA transfection. Transfection was performed, and cells were maintained in the presence of LAM. Supernatant was collected (without any purification steps) 2 days after transfection and concentrated with Amicon Ultra-0.5 ml 100 kDa centrifugal filters (Millipore, catalog no. UFC510096) to a volume of 200 μl. HBV DNA was then extracted using the QIAamp MinElute Virus Spin Kit (Qiagen, catalog no. 57704). DNA was eluted with 50 μl of buffer AVE and amplified by two PCRs before sequencing. The PCR products from both rounds were separated in a 1% TAE agarose gel and purified by gel extraction (Qiagen, catalog no. 28704). The first PCR used primers RU-O-26880 and RU-O-24242 to amplify near full-length HBV sequence in 100-μl volume using 50 μl of 2× KOD Hot Start Master Mix (EMD, catalog no. 71843), 20 μl of eluted DNA, and 15 pmol of each primer. The PCR was performed by (i) denaturation at 95°C for 1 min (one cycle); (ii) PCR at 95°C for 15 s, 62°C for 30 s, and 68°C for 60 s (25 cycles for no drug treatment and 32 cycles for 40 μM and 400 μM LAM treatment); and (iii) 68°C for 2 min and 10°C to hold. The PCR products were separated in a 1% TAE agarose gel and purified with the MinElute Gel Extraction kit (Qiagen, catalog no. 28604) and eluted in 15 μl of EB buffer. For the second PCR, primers RU-O-26265 and RU-O-26264 were used to amplify the RT region of HBV Pol using 50-μl volume and the following cycling conditions: (i) denaturation at 95°C for 1 min (one cycle); (ii) PCR at 95°C for 15 s, 62°C for 30 s, and 68°C for 30 s (12 cycles); and (iii) 68°C for 2 min and 10°C to hold. See table S3 for primer sequences. DNA from the second PCR was submitted to the CCIB DNA Core Facility, Massachusetts General Hospital (Cambridge, MA, USA) for high-throughput amplicon sequencing. Briefly, samples were sheared, ligated to Illumina-compatible adapters with unique barcodes, and sequenced from both ends on the MiSeq platform with 2 × 150 run parameters. Data were analyzed as described in the “Statistical analysis” section.

### Enriching resistant viral variants from DMS libraries

To enrich DMS mutations that confer LAM or BPV resistance, 2.5 × 10^5^ Huh-7.5-NTCP cells were seeded 2 days before pgRNA transfection as described above. To enrich LAM-resistant mutations, cells were treated with 400 μM LAM 24 hours before RNA transfection. To enrich BPV-resistant mutations, pgRNAs were cotransfected with 1:128 molar ratio of pgRNA:BPV or IRR ASO (control). Transfection was performed in triplicate wells of a six-well plate, and cells were harvested for DNA extraction 2 days after transfection.

HBV DNA was first amplified by PCR0 to obtain a near full-length HBV amplicon using primers listed in table S3 using KOD Xtreme Hot Start DNA Polymerase (Sigma-Aldrich, catalog no. 71975-3) and the following PCR conditions: 15 μl of 2× buffer, 1 μl of each primer (10 μM stock), 5 μl of 2 mM deoxynucleotide triphosphates, 1 μl of KOD Xtreme Pol, 5 μl of HBV DNA, and 2 μl of H_2_O. The PCR was performed by (i) denaturation at 94°C for 2 min (one cycle); (ii) PCR at 98°C for 10 s, 60°C for 30 s, and 68°C for 3 min; and (iii) 68°C for 2 min and 10°C to hold. The number of cycles was determined based on copy number from SYBR core amplicon qPCR results using the following equation: *y =* −1.864 * ln(*x*) + 43.336, where *x* = copy number/5 μl. The PCR products were separated in a 1% TAE agarose gel and purified with ADB (Zymo, catalog no. D4001-1-50) and a DNA Clean & Concentrator-5 column. PCR product was eluted in 10 μl of H_2_O and quantified by Qubit. The purified PCR0 product was used as template for PCR1 of the subamplicon sequencing method as described (*21*). The primers used for each of the three subamplicons are provided in table S3. PCR1 products were then gel isolated as above, Qubit-quantified, and diluted to be used as template for the barcoding and indexing PCR (PCR2). Templates were bottlenecked at 350,000 dsDNA molecules for each PCR2 reaction. To prepare sequencing libraries for the plasmid templates, we used 16 ng of plasmid as template directly for PCR1. Sequencing was performed at the Rockefeller University Genomics Resource Center. DMS libraries for the RT domain were sequenced by NovaSeq SP PE250. The 3N and 5N libraries surrounding the ASO binding site were sequenced by NextSeq PE150. Data were analyzed as described in the next section.

### Statistical analysis

Values presented are either means with error bars representing SEM with the number of replicates indicated in the figure legend or means with individual dots plotted. For dose-response curves, curve fitting was performed using the GraphPad Prism 9 software. R square values and 95% confidence intervals are indicated in the corresponding supplementary tables. For mouse experiments in Fig. 1, the *P* value was calculated by two-sided Fisher’s exact test using the GraphPad Prism 9 software.

Variants enriched from the T7 error–generated population were analyzed as follows: Amplicon sequences were mapped to the reference sequence using TopHat2 with a maximum of two mismatches allowed. Mapped sequences were read using the RSamtools package (*43*) and split into codons. Codons having one or more base with a quality score of <13 were ignored. For paired sample sets (one sample generated in the absence of LAM and one generated in the presence of 400 μM LAM), we performed one-sided Fisher’s exact tests for each possible substitution to obtain *P* values for enrichment in the presence of LAM. *P* values for substitutions from multiple independent paired sample sets were combined using the weighted z-method (*44*) and applied using the metap package (*45*). Source code for variant analysis can be obtained at github.com/ashleyacevedo/HBVseq_sub_enrichment. Sequences are deposited in the NCBI Sequence Read Archive, BioProject accession number PRJNA557489.

Variants enriched from DMS libraries were analyzed as follows: Fastq files were analyzed using dms2tools developed by J. Bloom. See here for a general introduction: https://github.com/jbloomlab/dms_tools2. The bcsubamp tool was used to correct for sequencing errors and quantify variants. The *P* values plotted in Fig. 4B were generated using EdgeR by comparing non-WT variants that were positively enriched by BPV (BPV versus IRR) at each position. *P* values for non-WT variants at each position were combined into a single value. The ggplot2 package was used to create the data visualizations in Fig. 4C. Variant counts were used as input to DESeq2 to generate the down-sampling table (table S4) for quality control. Data from table S4 were exported to Prism to generate fig. S7A. DESeq2 was also used to generate table S5, which lists the fold change and *P* value for variants enriched by BPV treatment. π = [−log_10_(*P* value) * log_2_FC] listed in table S5 combine the *P* value and fold change into a single value (*25*). π values in table S5 were exported to Prism to generate Fig. 4D and fig. S7B. Sequences are deposited in the NCBI Sequence Read Archive, BioProject accession number PRJNA557489.

## Notes

View/request a protocol for this paper from *Bio-protocol*.
