# Supplementary material for: An RNA-based system to study hepatitis B virus replication and evaluate antivirals
Source: Sci Adv. 2023 Apr 12;9(15):eadg6265. doi: 10.1126/sciadv.adg6265 (PMC10096565; doi:10.1126/sciadv.adg6265)
Supplement: Supplementary file 1 — Figs. S1 to S8 [file sciadv.adg6265_sm.pdf]

Science Advances
AAAS

Supplementary Materials for

An RNA-based system to study hepatitis B virus replication and evaluate antivirals

Yingpu Yu *et al.*

Corresponding author: William M. Schneider, wschneider@rockefeller.edu; Charles M. Rice, ricec@rockefeller.edu

Sci. Adv. **9**, eadg6265 (2023)
DOI: 10.1126/sciadv.adg6265

The PDF file includes:

Figs. S1 to S8

Other Supplementary Material for this manuscript includes the following:

Tables S1 to S6

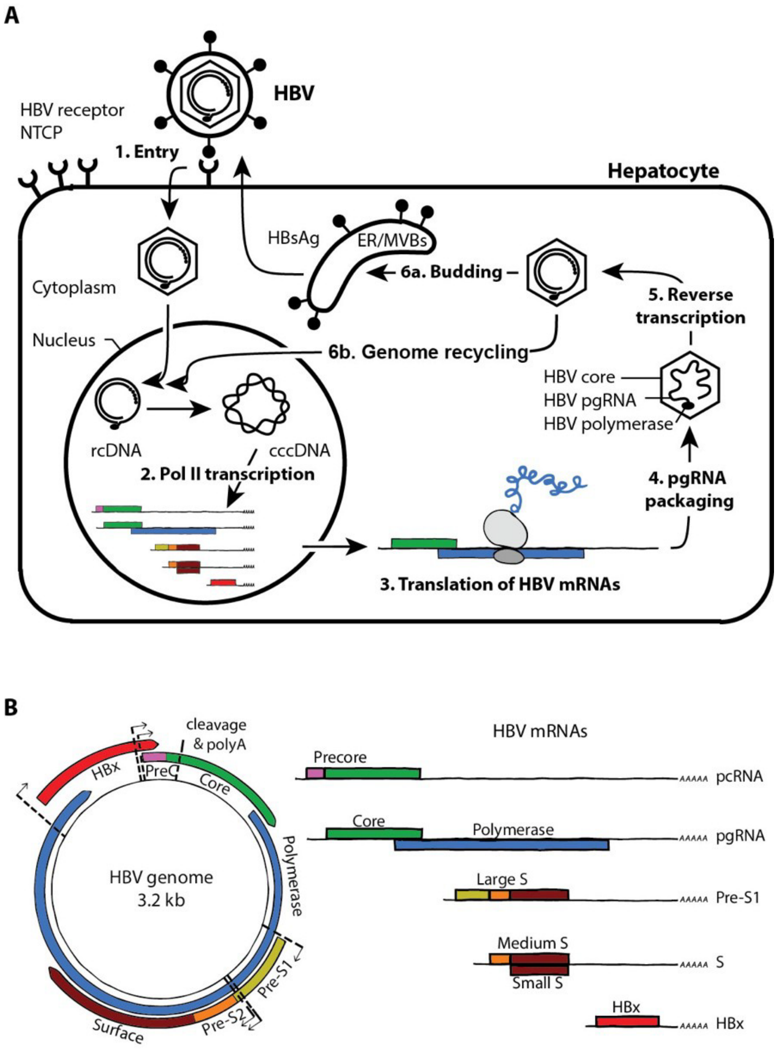

Fig. S1. HBV life cycle and mRNA transcripts. (**A**) HBV life cycle. HBV (1) enters hepatocytes via heparin sulfate proteoglycans (HSPGs) and sodium taurocholate cotransporting polypeptide (NTCP). In its encapsidated form, the genome is partially double-stranded, and upon entry to the nucleus, is converted from relaxed circular (rc) DNA to covalently closed circular (ccc) DNA. cccDNA is (2) transcribed by cellular Pol II to produce all viral mRNAs, which are then exported to the cytoplasm and (3) translated into viral proteins. Of these mRNAs, pgRNA, serves as the template for reverse transcription and encodes the core and polymerase proteins. The encapsidated pgRNA (4) is reverse transcribed (5) by HBV polymerase and can either (6a) associate with the viral surface antigens, HBsAg, and be exported from the cell, or (6b) "recycle" to the nucleus and

replenish cccDNA. ER, endoplasmic reticulum; MVBs, multivesicular bodies. (**B**) HBV genome and mRNA transcripts. Left, the highly compact, 3.2 kb double-stranded HBV DNA genome encodes multiple overlapping open reading frames (ORFs). Colored boxes indicate ORFs and arrows indicate Pol II transcriptional start sites. Right, the five HBV mRNAs. All transcripts terminate at the same cleavage and polyA addition signal.

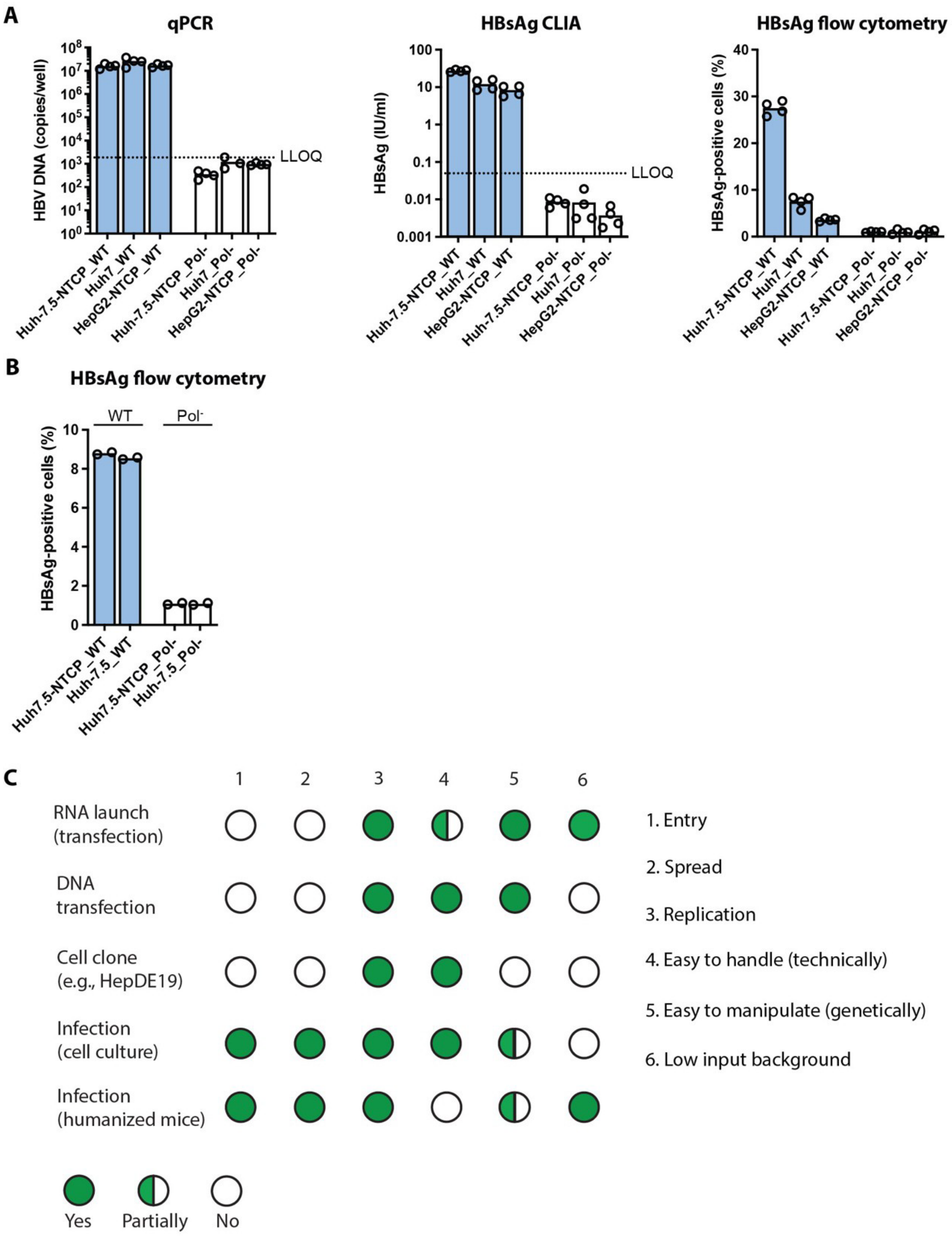

Fig. S2. RNA launch in several cell types and comparison with different systems. (**A**) Comparison of HBV DNA and HBsAg levels produced in three hepatoma cell lines commonly used for HBV research. Wild type (WT) or polymerase dead (Pol$^-$, YMHD) genotype A pgRNA

was transfected into each of the three cell lines (24 well plate). Two days post transfection, HBV DNA was quantified by qPCR. Six days post transfection, supernatants were collected for CLIA to quantify HBsAg and cells were fixed and stained to quantify the percentage of HBsAg+ cells. Graphs plot data from two independent experiments, each with two biological replicates. LLOQ = lower limit of quantification. LLOQ for CLIA = 0.05 IU/ml, which corresponds to less than one percent of the untreated control. The graph plots mean of four biological replicates with each replicate shown. (**B**) Comparison of Huh-7.5 vs Huh-7.5-NTCP cells. Six days post-transfection, cells were fixed and stained to quantify the percentage of HBsAg+ cells. Results indicate that the presence of the entry receptor, NTCP, does not affect the percentage of HBsAg-positive cells, supporting the conclusion that virus spread is minimal to non-existent under these conditions. Graph plots two biological replicates with each replicate shown. (**C**) Comparison of RNA launch method with various HBV experimental systems. Reasons for categorizing a method as "partially" are described here: for the RNA transfection method, RNA is more prone to degradation than DNA, and the method, therefore, requires more technical care. For the Infection with cell culture virus, the source of the virus will determine whether it is easy to genetically manipulate (e.g., whether it is from plasmid transfection or stable cell lines). Similarly, for infection in humanized mice, the source of the virus influences whether it can be readily manipulated (genetically) and the efficiency of infection.

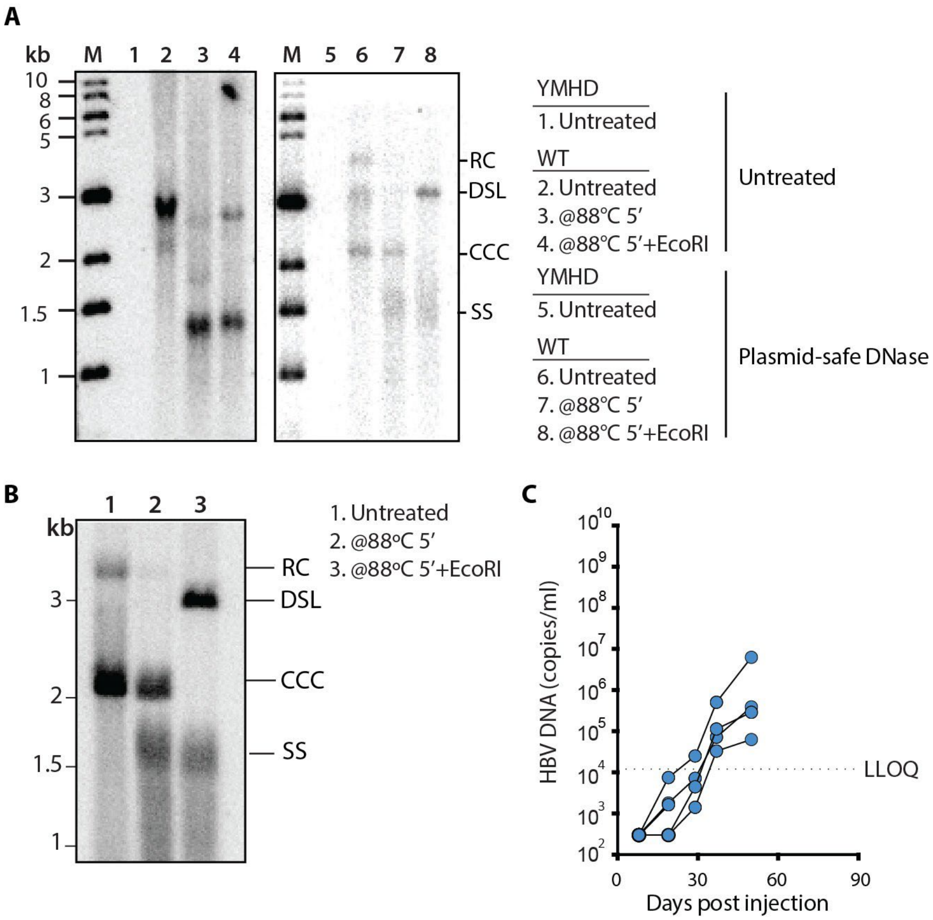

Fig. S3. HBV pgRNA in infectious. (**A**) Southern blot confirms rcDNA and cccDNA are produced in Huh-7.5-NTCP cells. The treatment conditions for the different lanes are indicated. Plasmid-safe DNase selectively degrades linear DNA while sparing rcDNA and cccDNA. kb, kilobase; YMHD, Pol⁻ HBV; RC, relaxed circular DNA; DSL, double-strand linear DNA; CCC, covalently closed circular DNA; SS, single-stranded DNA. (**B**) Southern blot detects HBV DNA in primary human hepatocytes isolated from a viremic huFNRG chimeric mouse infected with *in vitro*-transcribed HBV pgRNA. (**C**) HBV DNA copies per ml from mouse serum quantified by qPCR. Time post-inoculation with serum from HBV-positive mice infected with *in vitro*-transcribed HBV pgRNA is indicated. LLOQ = lower limit of quantification. N = 4.

A

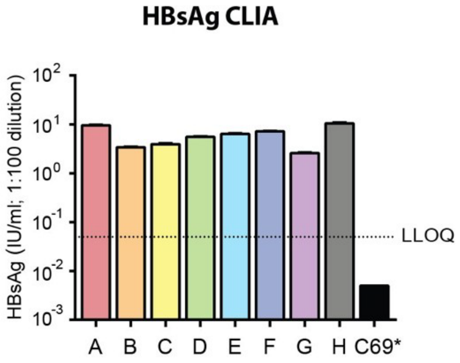

B

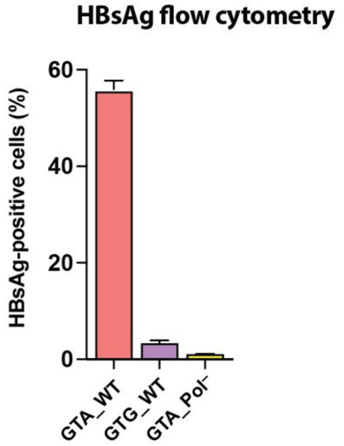

Fig. S4. HBsAg expression from HBV genotypes. (**A**) CLIA detects HBsAg from HBV genotypes A-H four days after transfecting cells with the plasmid templates used for *in vitro*-transcription. The results demonstrate that HBsAgs from each genotype are detectable by the CLIA kit, though with variable efficiency. The variation could be due to authentic differences in promoter/enhancer elements or differences in the ability of the antibodies in the kit to detect HBsAgs from the different genotypes. C69* was included as a negative control. This control plasmid (genotype A) encodes a stop codon in the HBs open reading frame at position 69 and does not produce detectable HBsAg. Supernatants were diluted 1:100 before detection to measure HBsAg in the same range as the experiment shown in **Fig. 2A**. Lower limit of quantification (LLOQ) = 0.05 IU/ml, which corresponds to less than one percent of the untreated control. N = 3

± SEM. **(B)** Intracellular HBsAg from gtA (WT and YMHD) and gtG were measured by flow cytometry. Few cells transfected with gtG pgRNA stained positive for HBsAg. While this demonstrates that HBsAg is produced from *in vitro*-transcribed gtG pgRNA, we conclude that multiple factors may contribute to the low HBsAg levels from gtG HBV: lower replication leading to fewer templates for HBsAg production, suboptimal detection by the CLIA kit or weaker promoter/enhancer elements, and, as previously reported, HBsAg aggregation in the ER. N = 3 ± SEM.

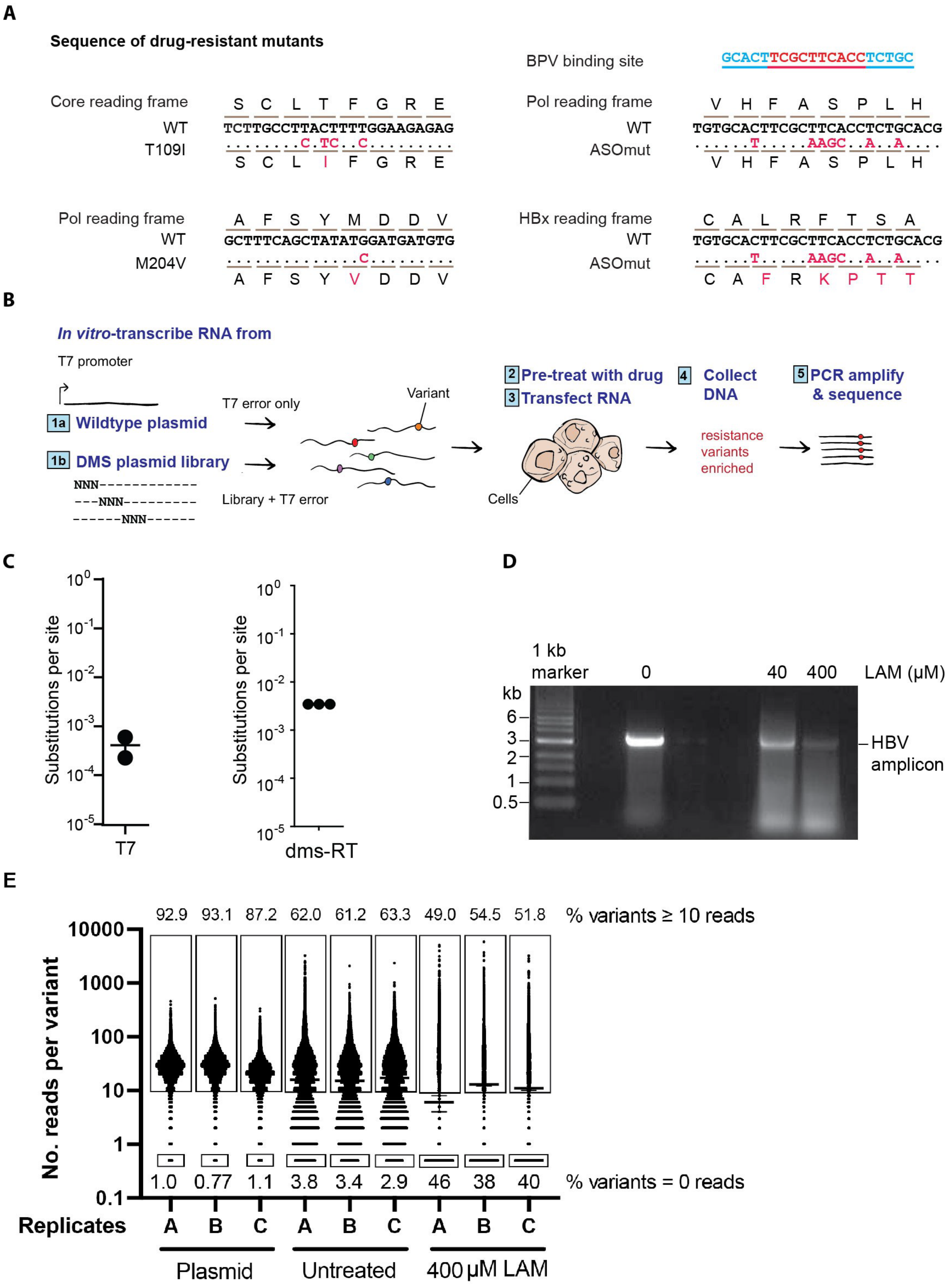
A
Sequence of drug-resistant mutants
Core reading frame
S C L T F G R E
WT
TCTTGCCTTACTTTTGGAAGAGAG
T109I
.......C.TC..C.........
S C L I F G R E
Pol reading frame
A F S Y M D D V
WT
GCTTTCAGCTATATGGATGATGTG
M204V
............C.........
A F S Y V D D V
BPV binding site
GCACTTCGCTTCACCTCTGC
Pol reading frame
V H F A S P L H
WT
TGTGCACTTCGCTTCACCTCTGCACG
ASOmut
.....T.....AAGC..A..A....
V H F A S P L H
HBx reading frame
C A L R F T S A
WT
TGTGCACTTCGCTTCACCTCTGCACG
ASOmut
.....T.....AAGC..A..A....
C A F R K P T T
B
In vitro-transcribe RNA from
T7 promoter
1a Wildtype plasmid
1b DMS plasmid library
NNN------------
---NNN---------
------NNN------
T7 error only
Library + T7 error
Variant
2 Pre-treat with drug
3 Transfect RNA
Cells
4 Collect DNA
resistance variants enriched
5 PCR amplify & sequence
C
Substitutions per site
T7
dms-RT
D
1 kb marker
0
40
400
LAM (μM)
kb
6
3
2
1
0.5
HBV amplicon
E
% variants ≥ 10 reads
92.9 93.1 87.2 62.0 61.2 63.3 49.0 54.5 51.8
No. reads per variant
10000
1000
100
10
1
0.1
% variants = 0 reads
1.0 0.77 1.1 3.8 3.4 2.9 46 38 40
Replicates
A B C A B C A B C
Plasmid
Untreated
400 μM LAM

Fig. S5. Selecting drug-resistant viral variants from *in vitro*-transcribed HBV pgRNA. (**A**) Sequence of the drug-resistant variants used in this study. Nucleotide and amino acid mutations are shown in red. We introduced silent mutants flanking the T109I mutations to uniquely mark this construct. Top right, the BPV binding sequence is colored as elsewhere in the manuscript: nucleotides that anneal to 2'MOE-modified wings of the ASO are colored blue; gap region is colored red. (**B**) Schematic describes a method to select and enrich mutations that confer resistance to HBV antivirals. From left, T7 bacteriophage RNA polymerase generates a diverse population of HBV pgRNA variants from either wildtype plasmid (1a) or a deep mutational scanning library of HBV variants (1b). These RNAs, transfected into cells in the presence of anti-HBV drugs, are subject to selection. HBV DNA is collected, amplified by PCR, and sequenced to identify drug-resistant variants. (**C**) Left, substitutions per site in *in vitro*-transcribed HBV pgRNA determined by CirSeq (n = 2); Right, substitutions per site in DMS plasmid library determined by subamplicon sequencing (n = 3). (**D**) 1% agarose gel of HBV DNA amplified two days post transfection from supernatants of cells untreated or treated with 40 or 400 μM LAM. (**E**) Panel shows the number of unique variants detected relative to the sequencing depth obtained. For the plasmid libraries, on average greater than 90% of all unique variants yielded 10 or more reads, with only ~1% of expected variants undetected. As expected, diversity is reduced in the reverse transcribed HBV DNA population as would be expected if some variants are unfit (untreated condition). Diversity is further reduced in the presence of LAM, consistent with strong selective pressure.

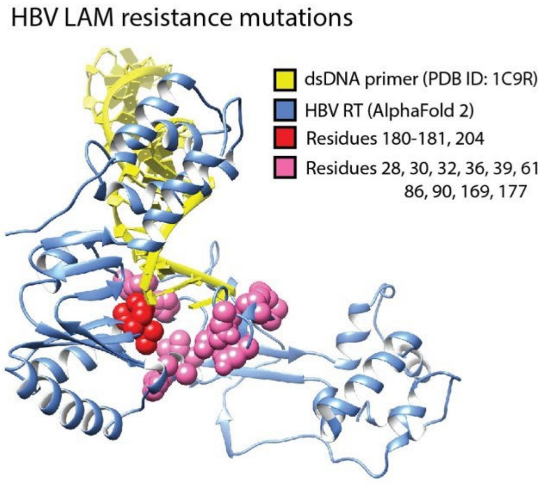

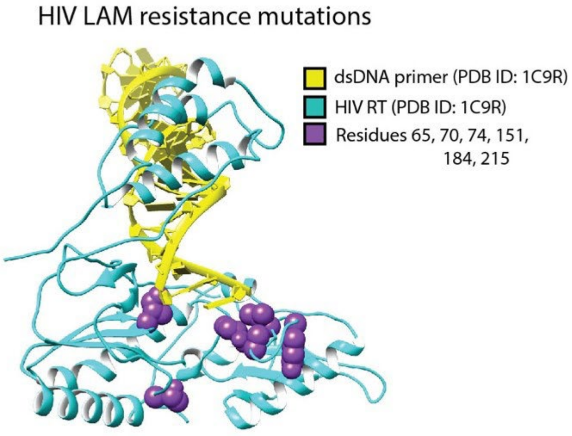

Fig. S6. LAM resistance mutations mapped onto HBV and HIV reverse transcriptase domain structures. Left, image is the same as **Fig. 3E** and is reproduced here to facilitate comparison. Right, crystal structure (PDB: 1C9R) of HIV reverse transcriptase domain with amino acids known to confer LAM resistance colored purple. Position 184 in HIV is homologous to position 204 in HBV. Known LAM resistance mutations in HIV RT correspond to spatially similar residues enriched in HBV RT in the presence of LAM.

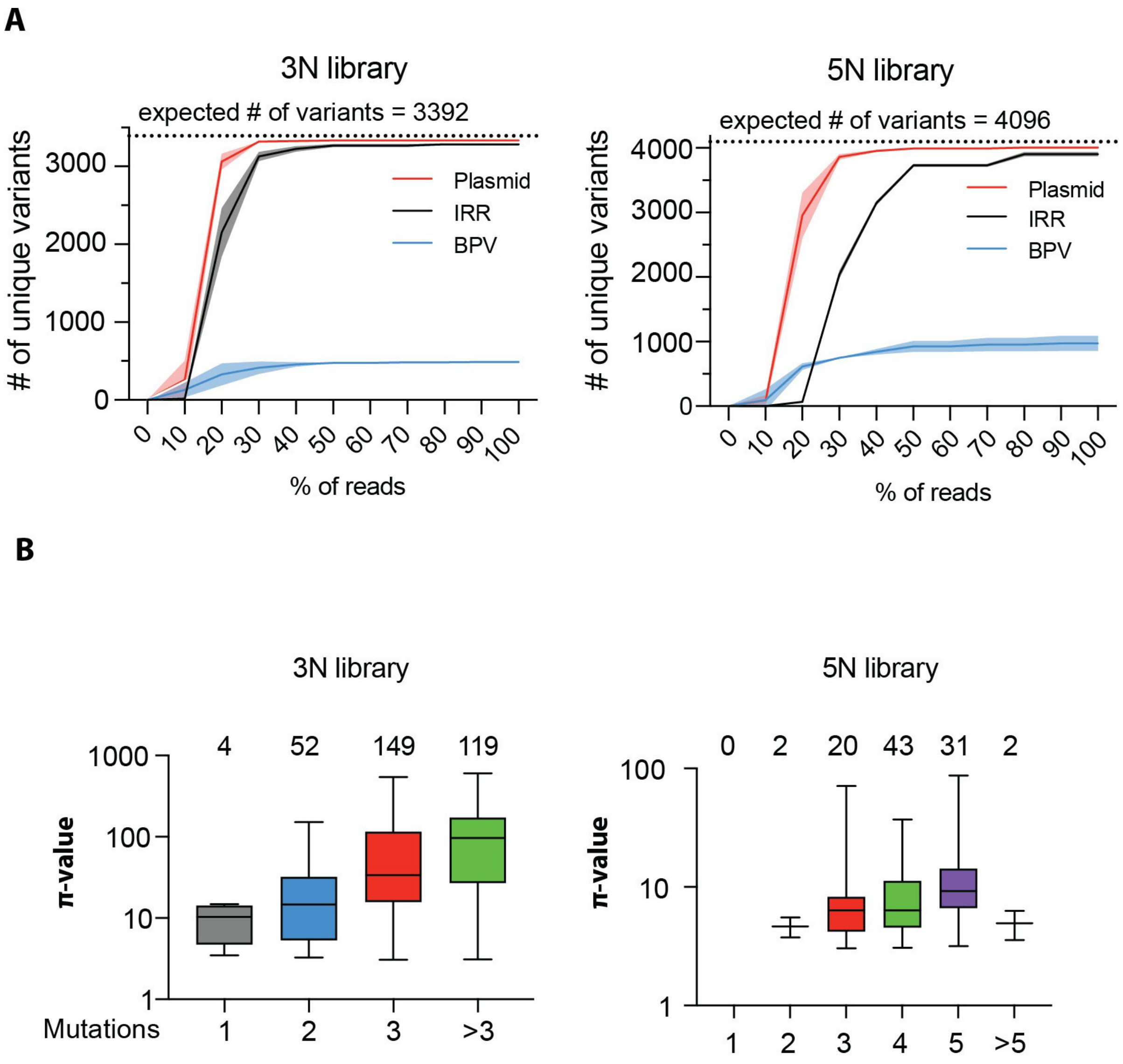

Fig. S7. 3N and 5N libraries with BPV selection. (**A**) Panel shows the number of unique variants detected relative to the sequencing depth obtained. Sequencing reads were down sampled at 10% increments. Dotted lines indicate the total number of expected variants. Results show that nearly all variants are detected with less than half of the sequencing reads acquired indicating that the libraries contain the expected diversity and the sequencing depth was sufficient. IRR-treated samples retain substantially more variants than BPV-treated samples, consistent with strong selective pressure exerted by BPV. (**B**) π-value for HBV individual sequences with the number of

mutations per sequence binned as indicated on the x-axis. The total number of sequences in each bin with $\pi \geq 3$ are indicated on top. Data from 3N library are identical to data presented in **Fig. 4D** and are reproduced here to facilitate comparison with 5N library.

A

Genotype	A
Number of sequences	855
Percentage coverage	98.2%

B

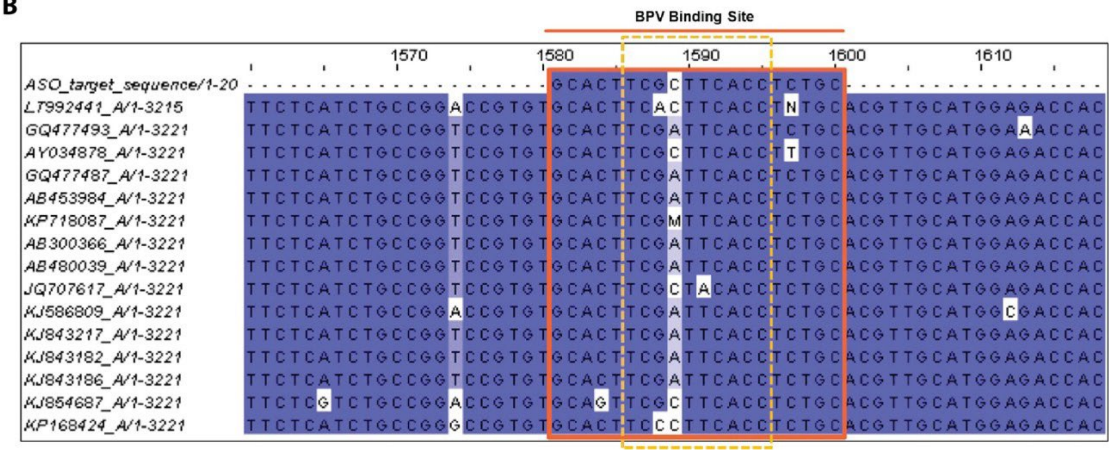

Fig. S8. Bioinformatics analysis of the BPV binding site in clinically relevant, publicly available HBV genome sequences. (**A**) The BPV target sequence is highly conserved. The number of HBV genotype A sequences available in HBVdb at the time of analysis (version: release 46.0 last updated 2018-12-16 (*28*)). A BLASTN search was carried out using BLAST version 2.6.0 using a word size of 5, using the BPV sequence as a query sequence, and the HBV sequences from HBVdb as the subject sequences. Percent coverage refers to the proportion of the available sequences that shared 100% sequence identity with the target BPV sequence. **(B)** Analysis of HBV genotype A sequences in the HBVdb that contained mismatches in the BPV target site. Multiple sequence alignment of the BPV target site and the n=15 HBV genotype A genomes in the HBVdb that contained mismatches in the BPV target site were generated using Muscle version 3.6. The BPV target site, which is 20 nucleotides in length, is highlighted in the red box; whereas the central 10 nucleotides within the BPV target sequence are highlighted by the orange dashed box. Of 855

genotype A HBV sequences present in the HBVdb at time of analysis, 9 (1.05%) contained the C9A variant.

Other Supplementary Materials for this manuscript include the following:

Tables S1 to S6

Table S1.
HBV genotypes

Table S2.
HBV inhibitor dose response

Table S3.
Primers

Table S4.
Variant countdown

Table S5.
Mutants enriched by BPV vs IRR

Table S6.
Genotype A C9A mutations in BPV binding site
